# Topology-Aware Multi-Omics Tensor Graph Clustering for Cancer Subtype Identification

**DOI:** 10.3390/biom16101402

**Published:** 2026-09-25

**Authors:** Hanrui Shi, Man-Fai Leung

**Affiliations:** 1 School of Information Sciences, University of Illinois Urbana-Champaign, Champaign, IL 61820, USA; hanrui0427@gmail.com; 2 School of Computing and Information Science, Faculty of Science and Engineering, Anglia Ruskin University, Cambridge CB1 1PT, UK

**Keywords:** clustering, cancer subtype identification, tensor graph learning, topology-aware similarity

## Abstract

Cancer subtype identification from multi-omics profiles is important for understanding tumor heterogeneity and advancing computational precision oncology. Various subtyping methods have been proposed, among which graph-based approaches have become a widely adopted paradigm by capturing patient-to-patient relationships from heterogeneous molecular profiles. However, the reliability of learned patient similarity graphs remains a critical challenge, as noisy or spurious edges induced by high-dimensional and heterogeneous omics measurements can impair subtype discrimination. To address this issue, we propose topology-aware tensor graph learning (TaTGL), an unsupervised multi-omics clustering framework for cancer subtyping. TaTGL first constructs omics-specific patient graphs using a topology-aware distance metric that integrates molecular-profile distance with shared-neighborhood consistency. Furthermore, to exploit both complementary and consistent information across different omics views, the resulting graphs are integrated through frequency-weighted t-SVD-based tensor graph learning, which captures high-order cross-omics structural consistency. Experimental studies on several cancer subtype datasets show that TaTGL achieves competitive or superior clustering performance on labeled datasets and identifies clinically relevant patient subgroups on survival-based datasets. These results demonstrate that the proposed method can discover molecularly coherent and clinically meaningful cancer subtypes from heterogeneous multi-omics profiles.

## 1. Introduction

Cancer is a highly heterogeneous disease, where tumors originating from the same tissue may differ substantially in their underlying biological mechanisms, clinical progression, therapeutic response, and survival outcomes. Cancer subtyping aims to stratify patients into clinically and biologically meaningful groups, and has, therefore, become a fundamental task in precision oncology [1,2]. With the rapid development of high-throughput sequencing technologies, comprehensive molecular profiles can now be characterized from multiple omics layers, including mRNA expression, DNA methylation, copy number variation, microRNA expression, proteomics, and other molecular modalities [1]. These multi-omics data provide complementary views of tumor biology and offer unprecedented opportunities to identify clinically relevant cancer subtypes. As a result, unsupervised clustering has become an effective paradigm for multi-omics cancer subtyping, as it can integrate heterogeneous molecular profiles and discover intrinsic patient groups without requiring predefined labels [3].

For this purpose, various multi-omics cancer subtyping methods have been proposed based on different integration strategies [4,5] (Table 1 summarizes representative multi-omics cancer subtyping methods by publication year, integration strategy, main advantages, and limitations or scope). A straightforward strategy is to concatenate features from multiple omics layers into a single matrix and then apply conventional clustering algorithms, such as k-means, spectral clustering, or non-negative matrix factorization [6,7]. However, these methods often suffer from increased dimensionality and may overlook modality-specific distributional differences. In contrast, consensus-based methods first analyze each omics layer independently and then combine the resulting clustering outputs through consensus or ensemble strategies [8,9]. Moreover, several joint modeling approaches have been developed for multi-omics cancer subtyping. For example, iCluster integrates multiple genomic data types through a joint latent variable model, in which different omics layers are assumed to share a common low-dimensional structure for sample clustering [10]. iClusterBayes extends this framework by introducing a fully Bayesian latent variable model and Bayesian variable selection, enabling integrative clustering while identifying omics features associated with latent subtype structures [11]. MOFA formulates multi-omics integration as an unsupervised factor analysis problem by decomposing heterogeneous omics data into a small number of latent factors that explain shared and view-specific sources of variation [12]. MOFA+ further improves the scalability and flexibility of MOFA, making it applicable to large-scale, multi-modal, and single-cell integration scenarios [13]. PIntMF applies penalized integrative matrix factorization with sparsity, non-negativity, and equality constraints to jointly cluster samples and select informative variables from multiple omics blocks [14]. Bayesian tensor factorization represents multi-omics data as a higher-order tensor and infers latent components within a Bayesian framework, thereby preserving cross-omics structural information for breast cancer subtyping [15].

Furthermore, graph-based methods have emerged as effective paradigms for modeling patient-to-patient relationships in cancer subtype discovery. For example, Similarity Network Fusion (SNF) constructs sample similarity networks for each omics modality and iteratively fuses them into a unified network for clustering [3]. NEMO extends similarity-based subtyping to partial multi-omics data by integrating available omics-specific similarity matrices [16]. CIMLR uses multiple kernels to estimate patient similarities and identify cancer subtypes [17].

In addition, recent advances in multi-view clustering have explored weighted tensor and topology-based graph learning. Gao et al. [18] use a weighted t-SVD nuclear norm to distinguish the contributions of singular values, while Xie et al. [19] learn high-order transition probabilities with an improved weighted tensor nuclear norm. Consensus graph learning jointly optimizes spectral embeddings and a low-rank tensor representation before learning a consensus graph in the embedding space [20]. More recently, CTCC constructs a cross-view topology graph using optimal-transport distances between view-specific bipartite graphs to extract consistent and complementary information [21]. These studies motivate distinguishing tensor representation learning and cross-view topology modeling from the within-view patient-edge reliability considered here. However, graph construction based primarily on pairwise distances or kernel similarities is sensitive to high-dimensional noise and may fail to adequately preserve local manifold structures [3,7,22,23]. In high-dimensional multi-omics data, two patients may appear close in terms of pairwise distance merely by chance but share few local neighbors, indicating an unreliable connection. Such noisy edges can blur subtype boundaries and reduce clustering robustness.

**Table 1:** Representative multi-omics cancer subtyping methods.

Method	Year	Category	Main Advantages	Limitations/Scope
iCluster [10]	2009	Joint latent-variable model	Shared representation and feature selection.	Linear latent structure; distributional assumptions.
SNF [3]	2014	Network fusion	Integrates complementary patient similarities.	Matched samples in the original formulation; neighborhood/kernel choices.
iClusterBayes [11]	2018	Bayesian latent- variable model	Mixed data types and Bayesian feature selection.	Likelihood/prior choices; MCMC convergence assessment.
CIMLR [17]	2018	Multiple-kernel learning	Learns similarities from multiple kernel scales.	Supplied kernel family; cluster-number selection.
NEMO [16]	2019	Neighborhood integration	Handles partial omics without imputation.	Requires shared omics for each patient pair.
PIntMF [14]	2022	Penalized matrix factorization	Sparse factors for clustering and feature selection.	Rank/penalty selection; nonconvex joint optimization.
BTF [15]	2022	Bayesian tensor factorization	Tensor-based multi-omics integration.	Reported application focuses on breast cancer.
MCLS [24]	2023	Latent-subspace learning	Projects incomplete samples without imputation.	Requires complete-omics samples; linear projections.
hMKL [25]	2023	Hierarchical kernel learning	Within-omics learning followed by cross-omics fusion.	Additive fusion without explicit interaction kernels.
MLMF [26]	2025	Multi-layer matrix factorization	Hierarchical integration of full and partial omics.	Depth/dimension choices; clustering follows fusion.
iMKL [27]	2025	Interaction-aware kernel learning	Models marginal and cross-omics interaction effects.	Chosen kernel construction; applications focus on renal cancer.
SIMKF [28]	2026	Survival-informed kernel fusion	Survival-guided selection and adaptive weighting.	Requires survival outcomes; separate prognostic validation needed.
TaTGL	This work	Topology-aware tensor graph learning	Shared-neighborhood graphs with frequency-weighted tensor regularization.	Matched cohorts; outcome-guided survival tuning; no external clinical validation.

To address this limitation, we propose a Topology-aware Tensor Graph Learning framework (TaTGL) for multi-omics cancer subtyping. As illustrated in Figure 1, cancer subtypes should be characterized by locally coherent patient groups rather than pairwise patient similarity alone. Specifically, a reliable connection between two patients should satisfy both molecular-profile similarity and shared-neighborhood consistency. Based on this motivation, we introduce a topology-aware distance metric that integrates pairwise molecular-profile distance with a shared-neighbor topological term. For two samples, the proposed metric rescales their distance using an exponential function determined by the overlap between their *k*-nearest-neighbor sets. When two patients share many local neighbors, the topological term reduces their effective distance and strengthens subtype-consistent connections. Conversely, when two patients have a small pairwise distance but share few neighbors, the metric avoids overemphasizing this potentially noisy edge. Combined with adaptive graph learning, the proposed metric can construct cleaner and more robust omics-specific patient similarity graphs. Furthermore, different omics layers contain both consensus subtype information and omics-specific molecular signals. Simple graph averaging may over-smooth view-specific patterns, whereas independent clustering may fail to exploit cross-omics complementarity [15,29,30]. To address this issue, we further couple topology-aware graph learning with a tensorized multi-view clustering model regularized by a frequency-weighted t-SVD-based tensor nuclear norm. The key idea of the proposed model lies in this coupling rather than weighted t-SVD regularization itself: shared patient neighborhoods guide within-view edge construction, whereas tensor regularization captures cross-view high-order dependence, jointly promoting locally reliable and cross-omics coherent patient graphs.

The main contributions of this work are summarized as follows:

- We propose a topology-aware local similarity learning strategy that jointly considers molecular-profile distance and shared-neighborhood consistency, thereby suppressing noise-induced graph edges in high-dimensional multi-omics patient graphs.
- We develop a unified model coupling topology-aware patient graph learning with frequency-weighted t-SVD regularization that integrates omics-specific patient graphs in a high-order tensor space, capturing cross-omics structural consistency while preserving informative complementary patterns across omics.
- We conduct comprehensive experiments on labeled and survival-based multi-omics cancer datasets, demonstrating that TaTGL achieves superior or competitive subtype discovery performance in terms of clustering accuracy, survival relevance, robustness, model stability, and biological interpretability.

The rest of this work is organized as follows. Section 2 presents the topology-aware local graph construction and frequency-weighted tensor graph learning model. Section 3 describes the optimization procedure. Section 4 reports the experimental setup, performance comparison, model analysis, ablation study, and biological analysis. Section 5 discusses the main findings and limitations, and Section 6 concludes this work.

## 2. Materials and Methods

In this section, we provide a detailed description of the proposed topology-aware tensor graph learning framework for unsupervised multi-omics cancer subtyping. Figure 2 summarizes the overall workflow of the proposed TaTGL.

### 2.1. Problem Definition

Let $X={\left\{X^{\left(v\right)}\right\}}_{v=1}^{V}$ denote a multi-omics dataset consisting of *V* omics views with *n* cancer samples, where $X^{\left(v\right)}\in R^{n\times d_{v}}$ is the feature matrix of the *v*-th omics modality, and $d_{v}$ is the number of molecular features in that view. The objective of cancer subtyping is to partition these *n* patients into *c* clinically meaningful subtypes.

### 2.2. The Proposed Method

We propose a topology-aware tensor graph clustering framework for unsupervised multi-omics cancer subtyping. The method contains three main components. First, for each omics view, we define the topology-aware patient distance metric, which integrates molecular-profile dissimilarity with local neighborhood consistency to reduce noise-induced graph edges. Based on this metric, we formulate an adaptive graph learning model to construct robust omics-specific patient similarity graphs. Finally, we introduce the tensorized multi-view clustering model with frequency-weighted t-SVD-based tensor nuclear norm regularization, which captures high-order cross-omics structural consistency while preserving modality-specific information.

**Topology-Aware Graph Learning:** In graph-based clustering, the quality of the learned sample relationship matrix S directly determines the clustering performance. However, most existing graph-based clustering methods face challenges in constructing reliable sample relationships from high-dimensional and heterogeneous multi-omics data. In particular, pairwise similarity measures based solely on direct distances are often sensitive to noise and may introduce spurious edges between samples that appear similar in the original feature space but are not supported by consistent local neighborhood structures. Such noisy graph connections can obscure intrinsic subtype boundaries and degrade the reliability of downstream clustering. To address this issue, we propose a topology-aware metric that strengthens sample pairs that are not only molecularly similar but also supported by shared local manifold neighborhoods, while avoiding overemphasis on pairwise associations that lack neighborhood consistency. In detail, for samples *i* and *j* in the *v*-th omics view, we first compute their patient-to-patient molecular similarity using the squared Euclidean distance:

(1) $${\tilde{d}}_{ij}^{\left(v\right)}={∥x_{i}^{\left(v\right)}-x_{j}^{\left(v\right)}∥}_{2}^{2},$$

where $x_{i}^{\left(v\right)}$ and $x_{j}^{\left(v\right)}$ denote the feature vectors of samples *i* and *j* in the *v*-th omics view, respectively. Then, we construct the *k*-nearest-neighbor set of each sample according to the initial distance. Let $N_{k}^{\left(v\right)}\left(i\right)$ denote the set of *k* nearest neighbors of sample *i* in the *v*-th omics view. The local topological overlap between samples *i* and *j* is defined as

(2) $$o_{ij}^{\left(v\right)}=\frac{\left|N_{k}^{\left(v\right)}\left(i\right)\cap N_{k}^{\left(v\right)}\left(j\right)\right|}{k}.$$

A larger value of $o_{ij}^{\left(v\right)}$ indicates that the two samples are embedded in more similar local neighborhood structures. Based on this topological overlap, the topology-aware distance is defined as

(3) $$d_{ij}^{\left(v\right)}={\tilde{d}}_{ij}^{\left(v\right)}\cdot \exp\left(-\lambda o_{ij}^{\left(v\right)}\right),$$

where λ>0 controls the strength of the topological correction. This metric has a clear biological and geometric interpretation. If two patients belong to the same subtype, they are expected to exhibit similar molecular profiles and share many local neighbors. In this case, $o_{ij}^{\left(v\right)}$ is large, and the exponential term reduces their effective distance, thereby strengthening subtype-consistent connections. In contrast, if two samples are close under the pairwise Euclidean distance but share few local neighbors, the exponential term remains close to one, preventing this potentially noisy edge from being overemphasized.

For each omics view, we learn an adaptive sample graph

(4) $$S^{\left(v\right)}\in R^{n\times n},$$

where $s_{ij}^{\left(v\right)}$ represents the learned similarity between samples *i* and *j* in the *v*-th omics view. Based on the proposed topology-aware metric, adaptive graph learning is formulated as

(5) $$\begin{array}{cc} \min_{S^{\left(v\right)}}\; & \sum_{i,j=1}^{n}d_{ij}^{\left(v\right)}s_{ij}^{\left(v\right)}+\gamma \sum_{i,j=1}^{n}{\left(s_{ij}^{\left(v\right)}\right)}^{2}\\ s.t.\; & s_{ij}^{\left(v\right)}\geq 0,\;\sum_{j=1}^{n}s_{ij}^{\left(v\right)}=1,\;s_{ii}^{\left(v\right)}=0. \end{array}$$

Substituting the topology-aware distance into the above objective, we have:

(6) $$\begin{array}{cc} \min_{s_{i}^{\left(v\right)}}\; & \sum_{j=1}^{n}{\tilde{d}}_{ij}^{\left(v\right)}\exp\left(-\lambda \frac{\left|N_{k}^{\left(v\right)}\left(i\right)\cap N_{k}^{\left(v\right)}\left(j\right)\right|}{k}\right)s_{ij}^{\left(v\right)}+\gamma \sum_{j=1}^{n}{\left(s_{ij}^{\left(v\right)}\right)}^{2}\\ s.t.\; & s_{ij}^{\left(v\right)}\geq 0,\;\sum_{j=1}^{n}s_{ij}^{\left(v\right)}=1,\;s_{ii}^{\left(v\right)}=0. \end{array}$$

The first term encourages each sample to connect with nearby samples under the topology-aware distance, while the second term controls the scale and smoothness of the learned similarities. The simplex constraint ensures that each row of $S^{\left(v\right)}$ forms a normalized similarity distribution, and the constraint $s_{ii}^{\left(v\right)}=0$ avoids trivial self-connections. As a result, the learned graph can preserve reliable subtype-consistent relationships while reducing noisy edges caused by high-dimensional omics measurements.

**Frequency-Weighted t-SVD-based Tensor Learning:** To further exploit complementary information across omics layers, we introduce a tensorized graph learning regularized by a frequency-weighted t-SVD-based tensor nuclear norm. This design aims to preserve high-order cross-omics structural consistency while retaining informative omics-specific graph patterns. Specifically, given the learned omics-specific patient similarity graphs ${\left\{S^{\left(v\right)}\right\}}_{v=1}^{V}$, we stack them into a third-order tensor: $S\in R^{n\times n\times V},$ where $S\left(:,:,v\right)=S^{\left(v\right)},\;v=1,\ldots ,V.$ This tensor representation naturally preserves the multi-omics structure of patient similarity graphs. The tensorized adaptive graph learning can be formulated as

(7) $$\begin{array}{cc} \min_{S}\; & \sum_{v=1}^{V}\left[\sum_{i,j=1}^{n}{\tilde{d}}_{ij}^{\left(v\right)}\exp\left(-\lambda \frac{\left|N_{k}^{\left(v\right)}\left(i\right)\cap N_{k}^{\left(v\right)}\left(j\right)\right|}{k}\right)s_{ij}^{\left(v\right)}+\gamma \sum_{i,j=1}^{n}{\left(s_{ij}^{\left(v\right)}\right)}^{2}\right]+\beta {∥S∥}_{⊛}\\ s.t.\; & S^{\left(v\right)}\geq 0,\;S^{\left(v\right)}1=1,\;s_{ii}^{\left(v\right)}=0,\;v=1,\ldots ,V, \end{array}$$

where ${∥S∥}_{⊛}$ denotes the t-SVD-based tensor nuclear norm, and β controls the strength of cross-omics low-rank regularization. However, the standard tensor nuclear norm penalizes all singular values uniformly in the Fourier domain. Such uniform shrinkage may excessively suppress large singular values that encode dominant subtype-related structures. To alleviate this issue, we replace the standard tensor nuclear norm with a frequency-weighted tensor nuclear norm. To reduce excessive shrinkage and better preserve dominant subtype-related components, we introduce the following frequency-weighted tensor nuclear norm:

(8) $$\Omega_{\mathrm{FW}-\mathrm{TNN}}\left(S\right)=\sum_{\ell =1}^{V}\omega_{\ell }\sum_{r}\phi \left(\sigma_{r}\left({\bar{S}}^{\left(\ell \right)}\right)\right),$$

where $\sigma_{r}\left({\bar{S}}^{\left(\ell \right)}\right)$ denotes the *r*-th singular value of the *ℓ*-th Fourier-domain frontal slice, $\omega_{\ell }$ is a frequency-specific weight, and ϕ(·) is a singular-value penalty function. Compared with the standard tensor nuclear norm, the proposed frequency-weighted regularization allows different Fourier components to contribute unequally to the low-rank constraint, thereby improving the preservation of informative cross-omics structures while suppressing noise-induced graph variations. In the optimization, the frequency weights at iteration *t* are evaluated from the current proximal input $M^{\left(t\right)}=S^{\left(t+1\right)}+Y^{\left(t\right)}/\rho_{t}$:

(9) $$\omega_{\ell }^{\left(t\right)}=\frac{1}{{∥{\hat{M}}_{\ell }^{\left(t\right)}∥}_{F}}.$$

where ${\hat{M}}_{\ell }^{\left(t\right)}$ denotes a Fourier-domain slice of $M^{\left(t\right)}$. The weights are slice-specific: a larger-norm slice receives a smaller penalty, whereas a smaller-norm slice receives stronger shrinkage. They remain fixed during the current Q-update and are recomputed from the new proximal input before the next Q-update. The tensor graph learning can be further rewritten as:

(10) $$\begin{array}{cc} \min_{S}\; & \sum_{v=1}^{V}\left[\sum_{i,j=1}^{n}{\tilde{d}}_{ij}^{\left(v\right)}\exp\left(-\lambda \frac{\left|N_{k}^{\left(v\right)}\left(i\right)\cap N_{k}^{\left(v\right)}\left(j\right)\right|}{k}\right)s_{ij}^{\left(v\right)}+\gamma \sum_{i,j=1}^{n}{\left(s_{ij}^{\left(v\right)}\right)}^{2}\right]\\ \; & +\beta \Omega_{\mathrm{FW}-\mathrm{TNN}}\left(S\right)\\ s.t.\; & S^{\left(v\right)}\geq 0,\;S^{\left(v\right)}1=1,\;s_{ii}^{\left(v\right)}=0,\;v=1,\ldots ,V. \end{array}$$

## 3. Optimization

In this section, we present the optimization procedure for the proposed method. The objective function contains two coupled components: the adaptive graph learning term for constructing omics-specific patient similarity graphs and the frequency-weighted t-SVD-based tensor nuclear norm regularization term for preserving high-order cross-omics consistency. Since the tensor nuclear norm is non-smooth and defined in the Fourier domain, directly optimizing the objective function is challenging. To address this issue, we adopt an alternating direction method of multipliers (ADMM) strategy by introducing an auxiliary tensor variable to decouple the graph learning and tensor regularization terms. The resulting problem can then be solved by alternately updating the omics-specific graph tensor, the auxiliary tensor variable, and the Lagrange multiplier until convergence. Specifically, we solve

(11) $$\begin{array}{cc} \min_{S}\; & \sum_{v=1}^{V}\left[\sum_{i,j=1}^{n}d_{ij}^{\left(v\right)}s_{ij}^{\left(v\right)}+\gamma \sum_{i,j=1}^{n}{\left(s_{ij}^{\left(v\right)}\right)}^{2}\right]+\beta \Omega_{\mathrm{FW}-\mathrm{TNN}}\left(S\right)\\ s.t.\; & S^{\left(v\right)}\geq 0,\;S^{\left(v\right)}1=1,\;s_{ii}^{\left(v\right)}=0,\;v=1,\ldots ,V. \end{array}$$

However, directly solving Equation (11) is challenging because the adaptive graph learning term is constrained by row-wise simplex constraints, while the frequency-weighted tensor nuclear norm is non-smooth and is defined in the Fourier domain. Here, we introduce an auxiliary tensor variable Q and impose the equality constraint S=Q. The optimization problem can be rewritten as:

(12) $$\begin{array}{cc} \min_{S,Q}\; & \sum_{v=1}^{V}\left[\sum_{i,j=1}^{n}d_{ij}^{\left(v\right)}s_{ij}^{\left(v\right)}+\gamma \sum_{i,j=1}^{n}{\left(s_{ij}^{\left(v\right)}\right)}^{2}\right]+\beta \Omega_{\mathrm{FW}-\mathrm{TNN}}\left(Q\right)\\ s.t.\; & S=Q,\\ \; & S^{\left(v\right)}\geq 0,\;S^{\left(v\right)}1=1,\;s_{ii}^{\left(v\right)}=0,\;v=1,\ldots ,V. \end{array}$$

In this way, we can solve Equation (12) using the alternating direction method of multipliers. The augmented Lagrangian function is defined as

(13) $$\begin{array}{cc} L(S,Q,Y)= & \sum_{v=1}^{V}\left[\sum_{i,j=1}^{n}d_{ij}^{\left(v\right)}s_{ij}^{\left(v\right)}+\gamma \sum_{i,j=1}^{n}{\left(s_{ij}^{\left(v\right)}\right)}^{2}\right]+\beta \Omega_{\mathrm{FW}-\mathrm{TNN}}\left(Q\right)\\ \; & +\langle Y,S-Q\rangle +\frac{\rho }{2}{∥S-Q∥}_{F}^{2}, \end{array}$$

where Y is the Lagrange multiplier tensor and ρ>0 is the penalty parameter. Thus, the variables S, Q, and Y are updated alternately until convergence.

### 3.1. Update of S

With Q and Y fixed, the S-subproblem can be written as

(14) $$\begin{array}{cc} \min_{S}\; & \sum_{v=1}^{V}\left[\sum_{i,j=1}^{n}d_{ij}^{\left(v\right)}s_{ij}^{\left(v\right)}+\gamma \sum_{i,j=1}^{n}{\left(s_{ij}^{\left(v\right)}\right)}^{2}\right]+\frac{\rho }{2}{∥S-Q+\frac{Y}{\rho }∥}_{F}^{2}\\ s.t.\; & S^{\left(v\right)}\geq 0,\;S^{\left(v\right)}1=1,\;s_{ii}^{\left(v\right)}=0,\;v=1,\ldots ,V, \end{array}$$

Then, we define

(15) $$B^{\left(v\right)}=Q^{\left(v\right)}-\frac{Y^{\left(v\right)}}{\rho }.$$

Then, for each omics view *v*, Equation (14) can be rewritten as

(16) $$\begin{array}{cc} \min_{S^{\left(v\right)}}\; & \sum_{i,j=1}^{n}d_{ij}^{\left(v\right)}s_{ij}^{\left(v\right)}+\gamma \sum_{i,j=1}^{n}{\left(s_{ij}^{\left(v\right)}\right)}^{2}+\frac{\rho }{2}\sum_{i,j=1}^{n}{\left(s_{ij}^{\left(v\right)}-b_{ij}^{\left(v\right)}\right)}^{2}\\ s.t.\; & S^{\left(v\right)}\geq 0,\;S^{\left(v\right)}1=1,\;s_{ii}^{\left(v\right)}=0. \end{array}$$

Since the constraints are imposed independently on each row of $S^{\left(v\right)}$, Equation (16) can be further decomposed into *n* row-wise subproblems. For the *i*-th row, we solve

(17) $$\begin{array}{cc} \min_{s_{i}^{\left(v\right)}}\; & \sum_{j=1}^{n}d_{ij}^{\left(v\right)}s_{ij}^{\left(v\right)}+\gamma \sum_{j=1}^{n}{\left(s_{ij}^{\left(v\right)}\right)}^{2}+\frac{\rho }{2}\sum_{j=1}^{n}{\left(s_{ij}^{\left(v\right)}-b_{ij}^{\left(v\right)}\right)}^{2}\\ s.t.\; & s_{ij}^{\left(v\right)}\geq 0,\;\sum_{j=1}^{n}s_{ij}^{\left(v\right)}=1,\;s_{ii}^{\left(v\right)}=0. \end{array}$$

After expanding the quadratic terms and removing the constants independent of $s_{i}^{\left(v\right)}$, Equation (17) is equivalent to

(18) $$\begin{array}{cc} \min_{s_{i}^{\left(v\right)}}\; & \left(\gamma +\frac{\rho }{2}\right)\sum_{j=1}^{n}{\left(s_{ij}^{\left(v\right)}\right)}^{2}+\sum_{j=1}^{n}\left(d_{ij}^{\left(v\right)}-\rho b_{ij}^{\left(v\right)}\right)s_{ij}^{\left(v\right)}\\ s.t.\; & s_{ij}^{\left(v\right)}\geq 0,\;\sum_{j=1}^{n}s_{ij}^{\left(v\right)}=1,\;s_{ii}^{\left(v\right)}=0. \end{array}$$

Following the simplex projection strategy, Equation (18) can be transformed into the following Euclidean projection problem:

(19) $$\min_{s_{i}^{\left(v\right)}}{∥s_{i}^{\left(v\right)}-\frac{\rho b_{i}^{\left(v\right)}-d_{i}^{\left(v\right)}}{2\gamma +\rho }∥}_{2}^{2},\;s.t.\;s_{i}^{\left(v\right)}\geq 0,\;{\left(s_{i}^{\left(v\right)}\right)}^{T}1=1,\;s_{ii}^{\left(v\right)}=0.$$

The Lagrangian function of Equation (19) is

(20) $$\begin{array}{cc} L\left(s_{i}^{\left(v\right)},\eta_{i},\phi_{i}\right)= & \frac{1}{2}{∥s_{i}^{\left(v\right)}-\frac{\rho b_{i}^{\left(v\right)}-d_{i}^{\left(v\right)}}{2\gamma +\rho }∥}_{2}^{2}\\ \; & -\eta_{i}\left({\left(s_{i}^{\left(v\right)}\right)}^{T}1-1\right)-\phi_{i}^{T}s_{i}^{\left(v\right)}, \end{array}$$

where $\eta_{i}$ is the Lagrange multiplier for the equality constraint, and $\phi_{i}\geq 0$ is the Lagrange multiplier vector for the non-negativity constraint. According to the Karush–Kuhn–Tucker conditions, the optimal solution of Equation (19) can be verified as

(21) $$s_{i}^{(v)*}={\left(\frac{\rho b_{i}^{\left(v\right)}-d_{i}^{\left(v\right)}}{2\gamma +\rho }+\eta_{i}1\right)}_{+},$$

where ${\left(\cdot \right)}_{+}=\max\left(\cdot ,0\right)$ is applied element-wise. The scalar $\eta_{i}$ is determined by the simplex constraint

(22) $${\left(s_{i}^{(v)*}\right)}^{T}1=1.$$

Considering the zero-diagonal constraint $s_{ii}^{\left(v\right)}=0$, the element-wise closed-form solution is written as

(23) $$s_{ij}^{(v)*}=\left\{\begin{array}{cc} {\left(\frac{\rho b_{ij}^{\left(v\right)}-d_{ij}^{\left(v\right)}}{2\gamma +\rho }+\eta_{i}\right)}_{+}, & j\neq i,\\ 0, & j=i, \end{array}\right.$$

where $\eta_{i}$ satisfies

(24) $$\sum_{j\neq i}{\left(\frac{\rho b_{ij}^{\left(v\right)}-d_{ij}^{\left(v\right)}}{2\gamma +\rho }+\eta_{i}\right)}_{+}=1.$$

Therefore, each row of $S^{\left(v\right)}$ can be updated by a closed-form simplex projection.

### 3.2. Update of Q

With S and Y fixed, the Q-subproblem is

(25) $$\min_{Q}\beta \Omega_{\mathrm{FW}-\mathrm{TNN}}\left(Q\right)+\frac{\rho }{2}{∥Q-\left(S+\frac{Y}{\rho }\right)∥}_{F}^{2}.$$

Let

(26) $$M=S+\frac{Y}{\rho }.$$

Then Equation (25) can be rewritten as

(27) $$\min_{Q}\beta \Omega_{\mathrm{FW}-\mathrm{TNN}}\left(Q\right)+\frac{\rho }{2}{∥Q-M∥}_{F}^{2}.$$

This is the proximal operator of the frequency-weighted tensor nuclear norm. It can be efficiently solved in the Fourier domain. First, we apply the fast Fourier transform along the third mode:

(28) $$\bar{M}=\mathrm{fft}\left(M,\left[\right],3\right).$$

For each frontal slice ${\bar{M}}^{\left(\ell \right)}$, we compute its singular value decomposition:

(29) $${\bar{M}}^{\left(\ell \right)}=U^{\left(\ell \right)}\Sigma^{\left(\ell \right)}{\left(V^{\left(\ell \right)}\right)}^{H},\;\ell =1,\ldots ,V.$$

At iteration *t*, the slice-specific weights ${\left\{\omega_{\ell }^{\left(t\right)}\right\}}_{\ell =1}^{V}$ are recomputed from the current proximal input according to Equation (9) and then held fixed during the Q-subproblem. When ϕ(x)=x, the frequency-weighted tensor nuclear norm leads to a weighted singular value thresholding operation:

(30) $${\bar{Q}}^{\left(\ell \right)}=U^{\left(\ell \right)}D_{\frac{\beta \omega_{\ell }}{\rho }}\left(\Sigma^{\left(\ell \right)}\right){\left(V^{\left(\ell \right)}\right)}^{H},$$

where $D_{\tau }\left(\cdot \right)$ denotes the singular value shrinkage operator:

(31) $$D_{\tau }\left(\Sigma \right)=\mathrm{diag}\left(\max(\sigma_{r}-\tau ,0)\right).$$

Therefore, β controls the overall regularization strength, whereas $\omega_{\ell }^{\left(t\right)}$ adjusts the shrinkage threshold across Fourier slices; setting all $\omega_{\ell }^{\left(t\right)}=1$ recovers the corresponding unweighted operation. Finally, the updated tensor Q is obtained by inverse FFT:

(32) $$Q=\mathrm{ifft}(\bar{Q},\left[\right],3).$$

### 3.3. Update of the Lagrange Multiplier

After updating S and Q, the Lagrange multiplier is updated by

(33) $$Y=Y+\rho \left(S-Q\right).$$

The penalty parameter can be adaptively increased as

(34) $$\rho =\min(\eta \rho ,\rho_{\max}),$$

where η>1 is an increasing factor and $\rho_{\max}$ is the maximum penalty value.

After convergence, we construct the final consensus graph by averaging the symmetrized omics-specific graphs:

(35) $$S^{*}=\frac{1}{V}\sum_{v=1}^{V}\frac{S^{\left(v\right)}+{\left(S^{\left(v\right)}\right)}^{⊤}}{2}.$$

The normalized graph Laplacian is

(36) $$L=I-D^{-1/2}S^{*}D^{-1/2},$$

where D is the degree matrix of $S^{*}$. Spectral clustering is then applied to L. Specifically, we take the eigenvectors corresponding to the *c* smallest eigenvalues, form a low-dimensional embedding matrix, and apply K-means to obtain the final cancer subtype labels. Algorithm 1 summarizes the complete optimization of **TaTGL**.
**Algorithm 1** Topology-aware tensor graph clustering**Require:** 
Multi-omics data $X={\left\{X^{\left(v\right)}\right\}}_{v=1}^{V}$, cluster number *c*, parameters k,λ,γ,β,ρ,ϵ,T,δ**Ensure:** 
Subtype labels y  1:Preprocess each omics matrix $X^{\left(v\right)}$.  2:**for** v=1,…,V **do**  3:      Compute Euclidean molecular-profile distance ${\tilde{d}}_{ij}^{\left(v\right)}={∥x_{i}^{\left(v\right)}-x_{j}^{\left(v\right)}∥}_{2}^{2}$.  4:      Construct *k*NN sets $N_{k}^{\left(v\right)}\left(i\right)$ and shared-neighbor scores $o_{ij}^{\left(v\right)}$.  5:      Compute $d_{ij}^{\left(v\right)}={\tilde{d}}_{ij}^{\left(v\right)}\exp\left(-\lambda o_{ij}^{\left(v\right)}\right)$.  6:      Initialize $S^{\left(v\right)}$ by adaptive graph learning.  7:**end for**  8:Stack ${\left\{S^{\left(v\right)}\right\}}_{v=1}^{V}$ into S and set Q=S; initialize Y=0 and ρ>0.  9:**for** t=1,…,T **do**10:      $S_{old}\leftarrow S$.11:      Update each $S^{\left(v\right)}$ by simplex projection using Equations (23) and (24).12:      Compute ${\left\{\omega_{\ell }^{\left(t\right)}\right\}}_{\ell =1}^{V}$ from the current proximal input using Equation (9).13:      Update Q by frequency-weighted t-SVD thresholding using Equations (30)–(32).14:      Update Y by Equation (33) and update ρ by Equation (34).15:      **if** ${∥S-S_{old}∥}_{F}/{∥S_{old}∥}_{F}<\delta$ **then**16:           **break**17:      **end if**18:**end for**19:Construct consensus graph$$S^{*}=\frac{1}{V}\sum_{v=1}^{V}\frac{S^{\left(v\right)}+{\left(S^{\left(v\right)}\right)}^{⊤}}{2}.$$20:Compute $L=I-D^{-1/2}S^{*}D^{-1/2}$.21:Apply spectral clustering on L to obtain y.22:**return** y

#### Complexity Analysis

We analyze the computational complexity of the proposed clustering. Let *n* be the number of patients, *V* be the number of omics views, *c* be the number of clusters, and *T* be the number of optimization iterations. Let $d_{v}$ be the feature dimension of view *v*, $D=\sum_{v=1}^{V}d_{v}$, *R* be the number of K-means restarts, and $I_{K}$ be the maximum number of K-means iterations per restart.

Before optimization, pairwise distance computation costs $O(n^{2}D)$. Row-wise sorting for nearest-neighbor selection costs $O(Vn^{2}\log n)$; sorting the topology-adjusted distances during graph initialization has the same order. Shared-neighborhood counts are computed by multiplying each symmetrized binary neighborhood matrix by its transpose. Writing $q_{i}^{\left(v\right)}$ for its node degrees, the sparse multiplication has an arithmetic cost of $O(C_{\mathrm{SNN}})$, where $C_{\mathrm{SNN}}=\sum_{v=1}^{V}\sum_{i=1}^{n}{\left(q_{i}^{\left(v\right)}\right)}^{2}$. Conversion to dense matrices and distance rescaling add $O(Vn^{2})$. The shared-neighborhood cost reduces to $O(Vnk^{2})$ when all degrees remain of order *k*.

In each iteration, updating S requires row-wise simplex projection for all omics-specific graphs, costing $O(JVn^{2})$ for the implemented iterative projection, where *J* is its bounded inner-iteration count. Updating Q involves frequency-weighted t-SVD in the Fourier domain. The n×n×V tensor is first rearranged to n×V×n, and the FFT is applied along the last dimension. Thus, the FFT and inverse FFT cost $O(n^{2}V\log n)$, and economy-size SVDs of the *n* frequency slices of size n×V cost $O(n^{2}V^{2})$ for V≤n. Computing the frequency weights adds $O(Vn^{2})$. These costs refer to the rearranged implementation; applying t-SVD directly to the unpermuted tensor would instead give $O(n^{2}V\log V+Vn^{3})$. The multiplier update only requires element-wise operations and costs $O(Vn^{2})$. Therefore, the per-iteration cost is $O(JVn^{2}+n^{2}V\log n+n^{2}V^{2})$.

After optimization, graph averaging costs $O(Vn^{2})$. Evaluating the normalization as general dense matrix products gives a conservative bound of $O(Vn^{3})$. The full spectral decomposition used in the code costs $O(n^{3})$, and K-means on the n×c spectral embedding costs $O(RI_{K}nc^{2})$. Accordingly, we have

$$O\;\left(\begin{array}{c} n^{2}D+Vn^{2}\log n+C_{\mathrm{SNN}}\\ \;+T\left(JVn^{2}+n^{2}V\log n+n^{2}V^{2}\right)+Vn^{3}+RI_{K}nc^{2} \end{array}\right).$$

Therefore, the overall computational complexity can be approximately expressed as $O(n^{2}D+Vn^{3})$, where the cubic term mainly arises from the spectral decomposition. Since the number of omics views *V* is typically small, the complexity is dominated by the sample size *n*.

## 4. Results

In this section, we evaluate the proposed TaTGL on several public multi-omics cancer datasets. The experiments are designed to assess its effectiveness from different aspects.

### 4.1. Experimental Setup

Datasets and Evaluation Metrics: We conduct experiments on several public multi-omics cancer datasets to evaluate the effectiveness of the proposed topology-aware tensor graph learning framework. These datasets cover two evaluation scenarios according to the available clinical annotations. Specifically, the first scenario includes datasets with available subtype or reference labels, namely BRCA (breast invasive carcinoma) and KIPAN (pan-kidney cohort). KIPAN combines KICH (kidney chromophobe), KIRC (kidney renal clear cell carcinoma), and KIRP (kidney renal papillary cell carcinoma). For these labeled datasets, we quantitatively evaluate clustering performance using standard external clustering metrics, including ACC, NMI, and Purity. Specifically,

$$\begin{array}{cc} \mathrm{ACC} & =\frac{1}{n}\max_{\pi }\sum_{i=1}^{n}1\left\{y_{i}=\pi \left({\hat{y}}_{i}\right)\right\},\\ \mathrm{NMI} & =\frac{I(Y;\hat{Y})}{\max\{H\left(Y\right),H\left(\hat{Y}\right)\}},\\ \mathrm{Purity} & =\frac{1}{n}\sum_{r}\max_{s}\left|C_{r}\cap G_{s}\right|. \end{array}$$

where $y_{i}$ and ${\hat{y}}_{i}$ are reference and predicted labels, π is a label permutation, *I* denotes mutual information, *H* denotes entropy, and $C_{r}$ and $G_{s}$ are predicted clusters and reference classes, respectively. All three metrics range from 0 to 1, with higher values indicating greater agreement with the reference labels.

The second scenario includes datasets without ground-truth subtype labels but with available survival information, namely AML (acute myeloid leukemia), BIC (breast invasive carcinoma), COAD (colon adenocarcinoma), GBM (glioblastoma multiforme), KRCCC (kidney renal clear cell carcinoma), LIHC (liver hepatocellular carcinoma), LSCC (lung squamous cell carcinoma), OV (ovarian serous cystadenocarcinoma), SARC (sarcoma), and SKCM (skin cutaneous melanoma). For these datasets, the discovered clusters are evaluated in terms of clinical relevance using Kaplan–Meier survival analysis and log-rank tests [31]. This setting allows us to assess whether the proposed method can identify patient groups with significantly different survival outcomes. The log-rank test assesses the null hypothesis of equal survival functions across the discovered groups; a smaller *p*-value indicates stronger evidence against this null, not a larger effect size. The $-\log_{10}\left(p\right)$ is a monotonic transformation, so higher values correspond to smaller *p*-values. Harrell’s C-index and its internal cross-validation protocol are defined in the subtype-derived survival risk analysis. The statistics of these datasets are summarized in Table 2. Overall, these datasets provide a comprehensive benchmark for evaluating clustering accuracy, survival relevance, and robustness across heterogeneous multi-omics profiles.

For the survival datasets, the number of clusters *c* is selected from the candidate range by retaining the value that yields the smallest log-rank *p*-value. For fair comparison, the same candidate range and selection criterion are applied to TaTGL and all compared methods for each survival cohort. Table 3 reports the selected cluster numbers for every method and cohort.

Compared methods: We compare the proposed TaTGL with representative multi-view and multi-omics clustering methods, including similarity-based, kernel-based, latent-variable, and factorization-based methods:

- SNF constructs patient similarity networks for different omics layers and iteratively fuses them into a unified network [3].
- CIMLR integrates multi-omics data through multiple kernel learning to infer cancer subtypes [17].
- iClusterBayes is a fully Bayesian latent-variable model for integrative clustering of multiple genomic data types [11].
- NEMO performs neighborhood-based multi-omics clustering and can handle partial multi-omics data [16].
- MCLS learns a shared latent subspace for multi-omics cancer subtyping and can also handle incomplete multi-omics data [24].
- hMKL adopts a hierarchical two-stage multi-kernel learning strategy that first learns omics-specific composite kernels and then fuses them by unsupervised multiple kernel learning [25].
- iMKL models omics–omics interactions by constructing marginal sample-similarity kernels and Hadamard-product interaction kernels under an unsupervised multi-kernel learning framework [27].
- MLMF is a multi-layer matrix factorization method for cancer subtyping using full and partial multi-omics datasets [26].
- SIMKF performs survival-informed multi-omics kernel fusion by combining survival-guided kernel selection with distribution-aware kernel weighting [28].

### 4.2. Implementation Details

In our method, the neighborhood size for graph initialization was fixed at k=15. The main model parameters γ and β, which control the quadratic graph regularization and frequency-weighted tensor regularization, respectively, were selected from {0.1,1,5,10,30,50}. The topology-scaling parameter was fixed at λ=1. For the ADMM optimization, the initial penalty parameter was set to $\rho_{0}=1$, and the maximum number of iterations was fixed at T=100. During optimization, the penalty parameter was updated by multiplying it by η=2 after each iteration; thus, $\rho_{0}$ denotes only the initial value, whereas ρ varies across iterations. The numerical stabilization constant used in the frequency-weight calculation was fixed at $\epsilon =10^{-8}$.

Therefore, in these survival experiments, only γ and β were treated as dataset-dependent model hyperparameters, whereas *k*, λ, $\rho_{0}$, η, *T*, and ϵ were kept fixed.

### 4.3. Results and Analysis

Table 4, Table 5 and Table 6 report the experimental results on labeled and survival-based multi-omics cancer datasets. All values are reported as the mean ± sample standard deviation. As shown in Table 4, TaTGL achieves the best or comparable performance on the labeled datasets, demonstrating its effectiveness in recovering subtype-consistent patient groups from multi-omics data. Table 5 and Table 6 further evaluate the clinical relevance of the discovered subtypes on survival datasets. TaTGL achieves the best or competitive results across different multi-omics cancer datasets. In particular, TaTGL obtains the best mean survival stratification on seven of the ten survival cohorts, including AML, BIC, GBM, KRCCC, LIHC, OV, and SKCM, with mean log-rank *p*-values of $7.4563\times 10^{-4}$, $8.1869\times 10^{-7}$, $1.0026\times 10^{-4}$, $8.3571\times 10^{-3}$, $6.3136\times 10^{-5}$, $1.5195\times 10^{-5}$, and $6.9097\times 10^{-5}$, respectively. Several baselines achieve the best mean result on specific datasets, including CIMLR on COAD and MCLS on LSCC and SARC. However, their performance is less consistent across datasets.

To further summarize survival-stratification reliability, Table 7 shows the total number of survival cohorts with statistically significant subtype separation under mean log-rank p<0.05. TaTGL is the only method that achieves significant survival separation on all ten datasets. In comparison, the strongest baselines, including CIMLR, NEMO, and MCLS, are significant on six datasets, followed by SNF on five datasets and iClusterBayes on three datasets. This result indicates that TaTGL not only obtains small *p*-values on individual cohorts but also provides more stable clinically relevant subtype discovery across heterogeneous cancer types. Overall, the experimental results demonstrate the effectiveness of the proposed TaTGL framework.

### 4.4. Model Analysis

We further conduct model analysis experiments to evaluate parameter robustness, noise robustness, and overall statistical performance across tasks. These experiments complement the main clustering and survival analyses by examining whether TaTGL depends heavily on a narrow parameter setting and how its performance changes under feature-level perturbations.

Parameter Sensitivity: Figure 3 shows the sensitivity of TaTGL with respect to γ and β on representative labeled and survival datasets under a shared parameter range. Here, γ controls the quadratic graph regularization and β controls the frequency-weighted tensor low-rank regularization in Equation (7). TaTGL maintains high clustering accuracy across multiple neighboring settings, indicating that the labeled clustering performance is not determined by a single isolated configuration. This suggests that graph regularization and tensor regularization can be tuned within a moderate range.

Robustness Analysis: Figure 4 further evaluates the robustness of TaTGL under feature-level perturbations. In this setting, zero-mean Gaussian noise is added to each omics feature, with the noise scale proportional to the corresponding feature-wise standard deviation. On KIPAN, TaTGL maintains nearly perfect clustering performance as the noise level increases from 0 to 0.30, with mean ACC decreasing only slightly from 0.9966 to 0.9925. On GBM, SKCM, and BIC, TaTGL also preserves strong survival stratification performance under moderate feature noise and remains among the best-performing methods even at the highest perturbation level. These results indicate that the proposed topology-aware tensor graph learning framework is robust to feature-level noise and can maintain stable subtype discovery performance on perturbed multi-omics profiles.

Statistical significance analysis: Table 8 summarizes the statistical comparison across all labeled clustering metrics and survival-clustering datasets. TaTGL achieves the lowest average rank and median rank, and obtains eleven best results and three second-best results across the sixteen evaluation tasks. The paired signed-rank tests further show that the ranking advantage of TaTGL over each baseline is statistically significant. These results indicate that the proposed method delivers more consistent performance across heterogeneous evaluation settings, rather than improving only on a small subset of datasets.

### 4.5. Ablation Study

We conduct ablation studies to evaluate the contribution of each major component in the proposed TaTGL framework. The complete TaTGL model is compared with four variants:

- TaTGL-w/o-FW-tSVD removes the frequency-weighted t-SVD-based tensor nuclear norm and uses standard t-SVD thresholding instead.
- TaTGL-w/o-TPGL replaces the proposed topology-aware graph learning module with a conventional Euclidean adaptive graph construction strategy.
- TaTGL-w/o-LocalManifold removes local manifold modeling and constructs dense global Gaussian similarity graphs.
- TaTGL-w/o-Tensor removes the tensor graph learning module and directly averages the view-specific graphs for clustering.

Table 9 and Table 10 report the ablation results on the labeled cancer subtype datasets and the unlabeled survival datasets, respectively. On KIPAN, the complete TaTGL model achieves the best ACC, NMI, and Purity, demonstrating the joint effectiveness of topology-aware graph construction and frequency-weighted tensor fusion. On BRCA, TaTGL obtains the highest ACC and Purity, although the variant without TPGL achieves a slightly higher NMI, suggesting that different metrics may emphasize different aspects of subtype agreement. Removing frequency-weighted t-SVD regularization degrades performance on KIPAN and weakens ACC and Purity on BRCA, confirming the benefit of adaptive frequency-domain regularization. Replacing TPGL with Euclidean adaptive graphs or removing local manifold modeling leads to clear degradation on KIPAN and most survival datasets, indicating the importance of local topological consistency for reliable graph construction. In addition, removing the tensor learning module substantially reduces KIPAN performance and weakens survival stratification, verifying the necessity of high-order cross-omics graph fusion. For the unlabeled survival datasets, the complete TaTGL model achieves the smallest log-rank *p*-values on LIHC, BIC, and SKCM. Overall, the ablation results demonstrate that the topology-aware distance metric, adaptive graph learning, and frequency-weighted tensor graph regularization are all important to the effectiveness of TaTGL.

### 4.6. Biological Analysis

In this subsection, we conduct biological analyses from multiple perspectives to evaluate the interpretability and clinical relevance of the subtypes discovered by TaTGL.

Survival Analysis of Identified Subtypes: To evaluate the clinical relevance of the discovered subtypes, we conduct Kaplan–Meier survival analysis on representative cancer datasets. As shown in Figure 5, we compare the survival curves generated by TaTGL with those obtained by iMKL and MCLS on AML, BIC, OV, and GBM. The log-rank *p*-value is used to assess whether the identified patient subgroups exhibit significantly different survival outcomes. From the results, TaTGL identifies survival-discriminative patient subgroups across the four datasets. For example, TaTGL achieves significant log-rank *p*-values of $7.46\times 10^{-4}$ on AML, $8.19\times 10^{-7}$ on BIC, $1.52\times 10^{-5}$ on OV, and $1.00\times 10^{-4}$ on GBM, indicating clear survival separation among the discovered subtypes. Although iMKL and MCLS also show significant survival separation on specific datasets, their performance is less consistent across cohorts. Overall, the Kaplan–Meier analysis demonstrates that the subtypes identified by TaTGL are not only numerically separable but also associated with distinct clinical outcomes. These results indicate that the proposed method is able to identify clinically meaningful cancer subtypes from heterogeneous multi-omics data.

Subtype-Associated Gene Heatmap Analysis: Figure 6 shows the representative subtype-associated gene heatmaps of the discovered subtypes on AML, LSCC, SARC, and SKCM. The samples are ordered according to the TaTGL clustering results, while each row corresponds to a selected gene normalized by row-wise z-score. The displayed features were obtained from the gene-expression view of the TCGA-derived Multi-Omics Cancer Benchmark described in Section 4.1. Selection was performed after clustering and did not affect TaTGL optimization or the clustering solution. For each subtype, features were ranked by the difference between its mean row-standardized expression and the average of the mean expression values of the remaining subtypes. A fixed number of the highest-ranked features per subtype were selected.

From the heatmaps, clear block-wise expression patterns can be observed across different cancer datasets. Specifically, in AML, the selected genes associated with different subtypes show distinct relative expression patterns, indicating that the discovered patient groups have subtype-specific molecular characteristics. Similar patterns can also be observed in LSCC, where different groups of selected genes exhibit heterogeneous expression profiles across the identified subtypes. These results suggest that the subtypes identified by TaTGL are associated with coherent and subtype-specific molecular patterns.

Subtype-aware Similarity Structure Analysis: To further examine whether TaTGL can learn subtype-consistent patient relationships, we visualize the consensus matrices on BIC, GBM, and AML. As shown in Figure 7, samples are ordered according to the subtypes identified by TaTGL. Clear block-wise structures can be observed along the diagonal, indicating that patients assigned to the same subtype are stably grouped together across different datasets. Specifically, on AML, BIC and GBM, TaTGL produces well-separated block structures, where intra-subtype consensus probabilities are higher than inter-subtype probabilities. These results further support the effectiveness of TaTGL in identifying coherent cancer subtypes from multi-omics profiles.

Subtype-Derived Survival Risk Analysis: In the subtype-derived survival risk analysis, we further quantified whether the discovered subtypes provide subtype-level prognostic risk ordering. This analysis is consistent with survival-aware subgroup identification, where subtype discovery is evaluated by both molecular separation and prognostic discrimination [32]. Instead of estimating risk from the number of events and accumulated person-time, subtype membership is entered as a categorical covariate in a Cox proportional hazards model [33]. For subtype *s*, we report the Cox hazard ratio relative to the estimated lowest-risk subtype:

$${\mathrm{HR}}_{s}=\exp\;\left(\theta_{s}-\min_{s^{\prime }}\theta_{s^{\prime }}\right),$$

where $\theta_{s}$ is the fitted subtype coefficient. Thus, the lowest-risk subtype has HR=1. This formulation does not use a pseudo-count and is invariant to the unit in which follow-up time is recorded.

To avoid the potential optimism of an in-sample C-index, we report Harrell’s C-index using five-fold cross-validation [34]. The folds are stratified by subtype and event status. In each fold, Cox coefficients are estimated from the other four folds and applied to patients in the held-out fold. Concordant and comparable pairs are evaluated only within the held-out folds and pooled across the five folds:

$$C=\frac{N_{\mathrm{concordant}}+0.5N_{\mathrm{tied}}}{N_{\mathrm{comparable}}}.$$

A C-index of 0.5 indicates chance-level risk ordering, whereas a value of 1 indicates perfect concordance. Therefore, this analysis complements Kaplan–Meier and log-rank tests by assessing not only whether the discovered subtypes exhibit distinct survival curves, but also whether they induce an interpretable ordering of subtype-level survival risk. As shown in Figure 8, we compare the relative subtype-risk profiles discovered by TaTGL with those obtained by CIMLR and iMKL on AML, KRCCC, and BIC. The five-fold cross-validated C-indices of TaTGL are 0.610, 0.639, and 0.835 on these datasets, respectively. From the results, TaTGL identifies subtypes with distinguishable survival risks across the three datasets. For example, on AML, the subtypes discovered by TaTGL show clear differences in relative risk values, indicating that the identified patient groups are associated with different levels of survival risk. Similar observations can be found on KRCCC and BIC, where TaTGL separates patients into subtypes with distinct risk patterns. In contrast, the competing methods often produce subtypes with smaller risk differences or less consistent subtype-risk ordering. Together with the Kaplan–Meier survival analysis, these results indicate that TaTGL can discover patient groups with distinct survival outcomes and risk profiles.

## 5. Discussion

The results suggest that incorporating local topology into patient graph construction can reduce unreliable pairwise associations in high-dimensional multi-omics data. Compared with methods that directly fuse similarity matrices or kernels, TaTGL first improves the quality of omics-specific patient graphs and then integrates them through tensor graph learning. This design is consistent with the biological expectation that cancer subtypes should be supported by coherent local neighborhoods across molecular layers rather than by isolated pairwise similarities.

The study has several limitations. First, the analysis is based on public retrospective multi-omics cohorts, and prospective clinical validation was not performed. Second, the biological interpretation focuses on survival separation, marker-gene patterns, learned similarity structures, and subtype-level risk profiles; further experimental validation would be needed to confirm the mechanistic role of specific molecular markers. Third, the current implementation relies on parameter selection for graph construction and tensor regularization, although the ablation and sensitivity analyses indicate that the main components contribute consistently across datasets.

## 6. Conclusions

In this paper, we proposed TaTGL, a topology-aware tensor graph learning framework for unsupervised multi-omics cancer subtyping. By incorporating shared-neighborhood consistency, TaTGL constructs more reliable patient similarity graphs and reduces noisy pairwise connections. Moreover, the tensor graph learning module captures high-order complementary information across multiple omics views. Experiments on public multi-omics cancer datasets show that TaTGL achieves superior or competitive clustering performance on labeled datasets and identifies survival-distinct subtypes on unlabeled datasets. Ablation studies, algorithmic diagnostics, and biological analyses further demonstrate the effectiveness, robustness, optimization stability, and clinical relevance of the proposed method.

## Figures and Tables

**Figure 1:**
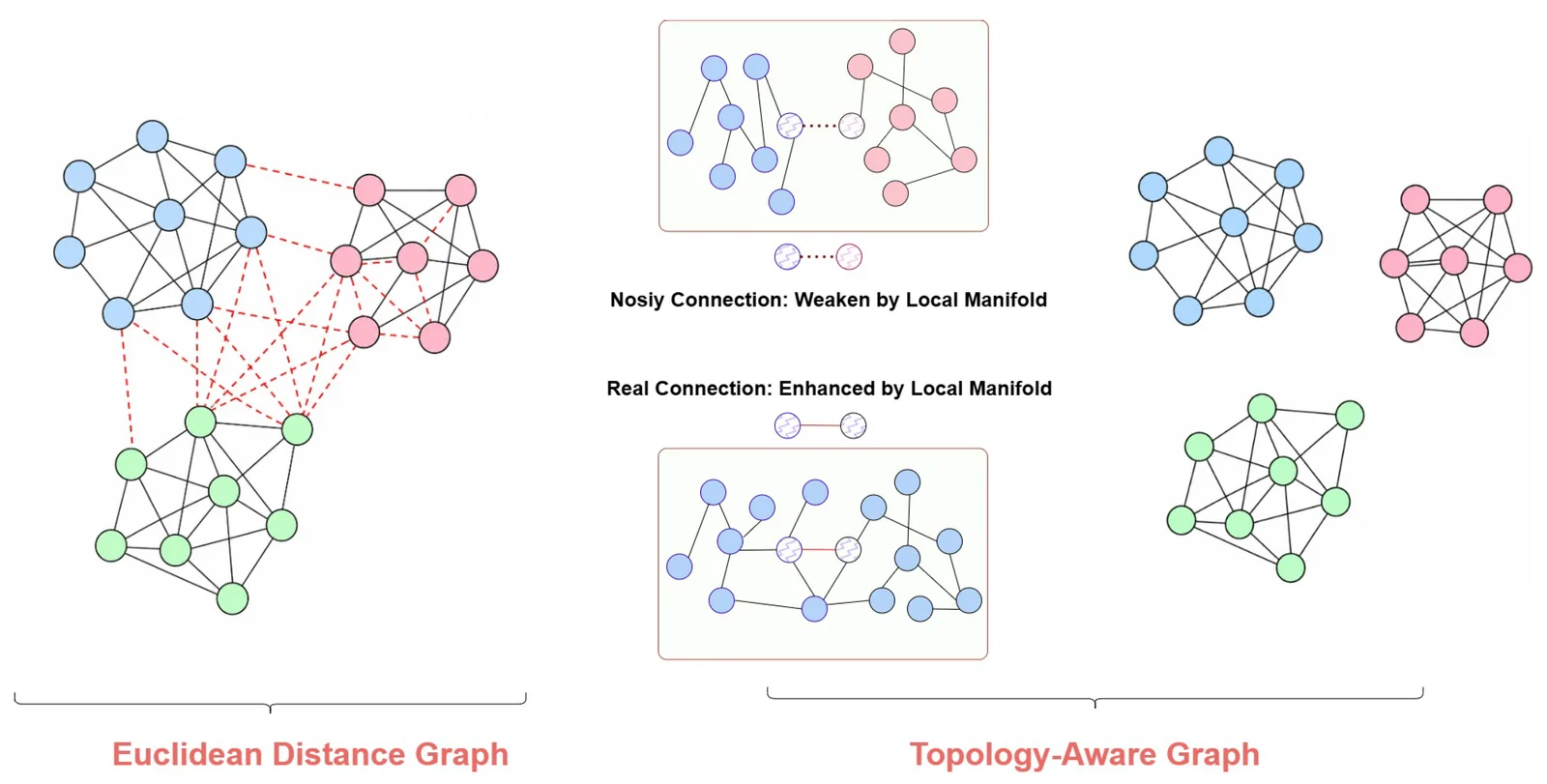
Motivation of the proposed topology-aware similarity metric. In the left and right panels, blue, pink, and green nodes represent patients from different groups. Gray solid lines indicate within-group connections, whereas red dashed lines in the left panel indicate noisy cross-group connections. The left panel shows a Euclidean-distance graph with noisy cross-group edges; the middle illustrates shared-neighborhood consistency, and the right shows the resulting topology-aware graph schematically. In high-dimensional multi-omics data, direct pairwise similarities may introduce noisy cross-subtype connections between samples from different local manifolds. The proposed metric uses shared-neighborhood consistency to weaken unsupported noisy links and enhance reliable within-manifold connections, resulting in cleaner patient graphs for subtype discovery.

**Figure 2:**
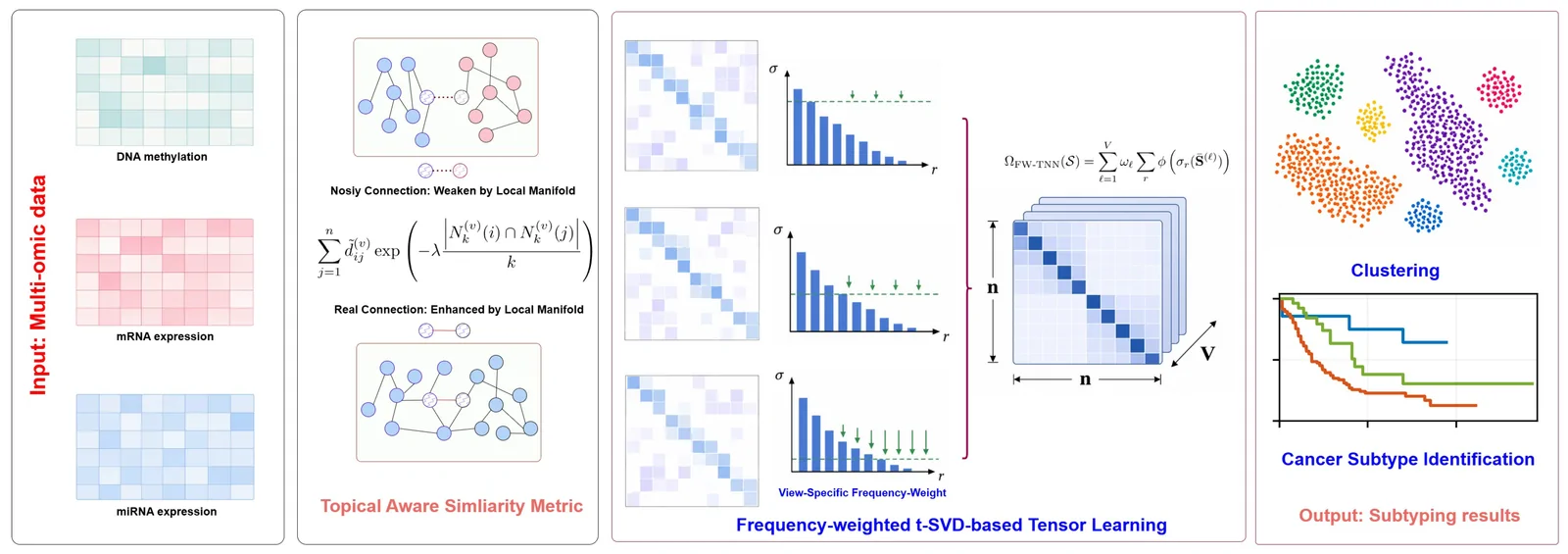
Overall workflow of TaTGL for multi-omics cancer subtyping. From left to right, the diagram shows the multi-omics inputs, topology-aware graph construction, frequency-weighted tensor graph learning, and subtyping outputs. The input heatmap colors distinguish different omics modalities. In the graph schematics, different node colors distinguish patient groups, and dashed links indicate noisy cross-group connections. TaTGL constructs omics-specific graphs using a topology-aware similarity metric that weakens noisy cross-manifold connections and enhances reliable within-manifold connections. The learned graphs are finally integrated by frequency-weighted t-SVD-based tensor learning, where view-specific frequency weights regularize the singular values of graph slices to capture high-order cross-omics structural consistency for subtype discovery. In the singular-value diagrams, blue bars represent singular values, while green arrows and dashed horizontal lines schematically illustrate the view-specific weighted shrinkage operation. Different colors in the clustering output distinguish the resulting patient groups, and the colored survival curves illustrate downstream survival evaluation of the identified subtypes.

**Figure 3:**
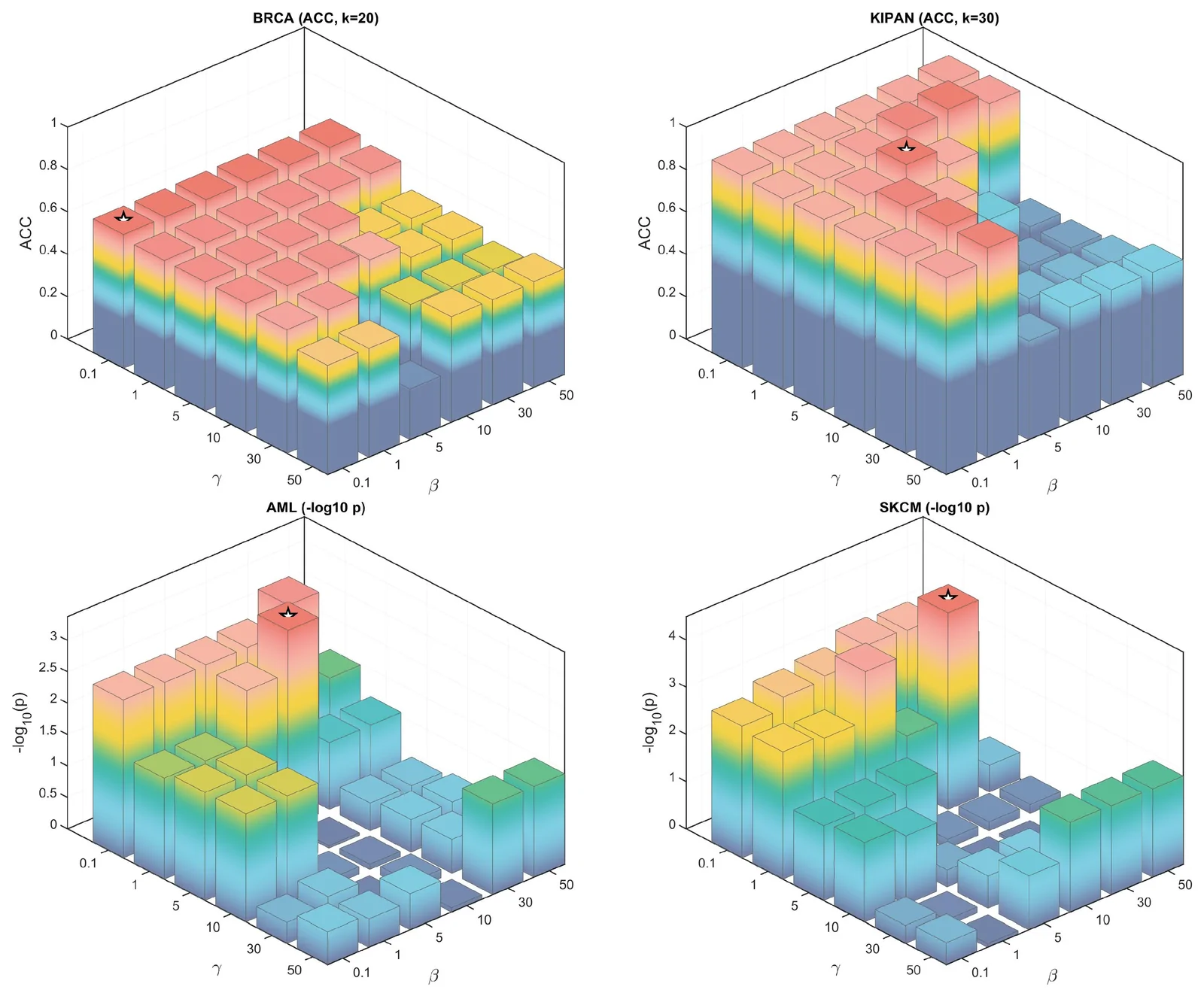
Parameter sensitivity analysis of TaTGL on BRCA, KIPAN, AML, and SKCM under the same γ/β grid {0.1,1,5,10,30,50}. The labeled datasets BRCA and KIPAN are evaluated by ACC, while AML and SKCM are evaluated by $-\log_{10}\left(p\right)$ based on the log-rank test. Bar colors visually encode the corresponding performance values, with warmer colors indicating higher values. The star in each panel indicates the parameter combination achieving the best performance.

**Figure 4:**
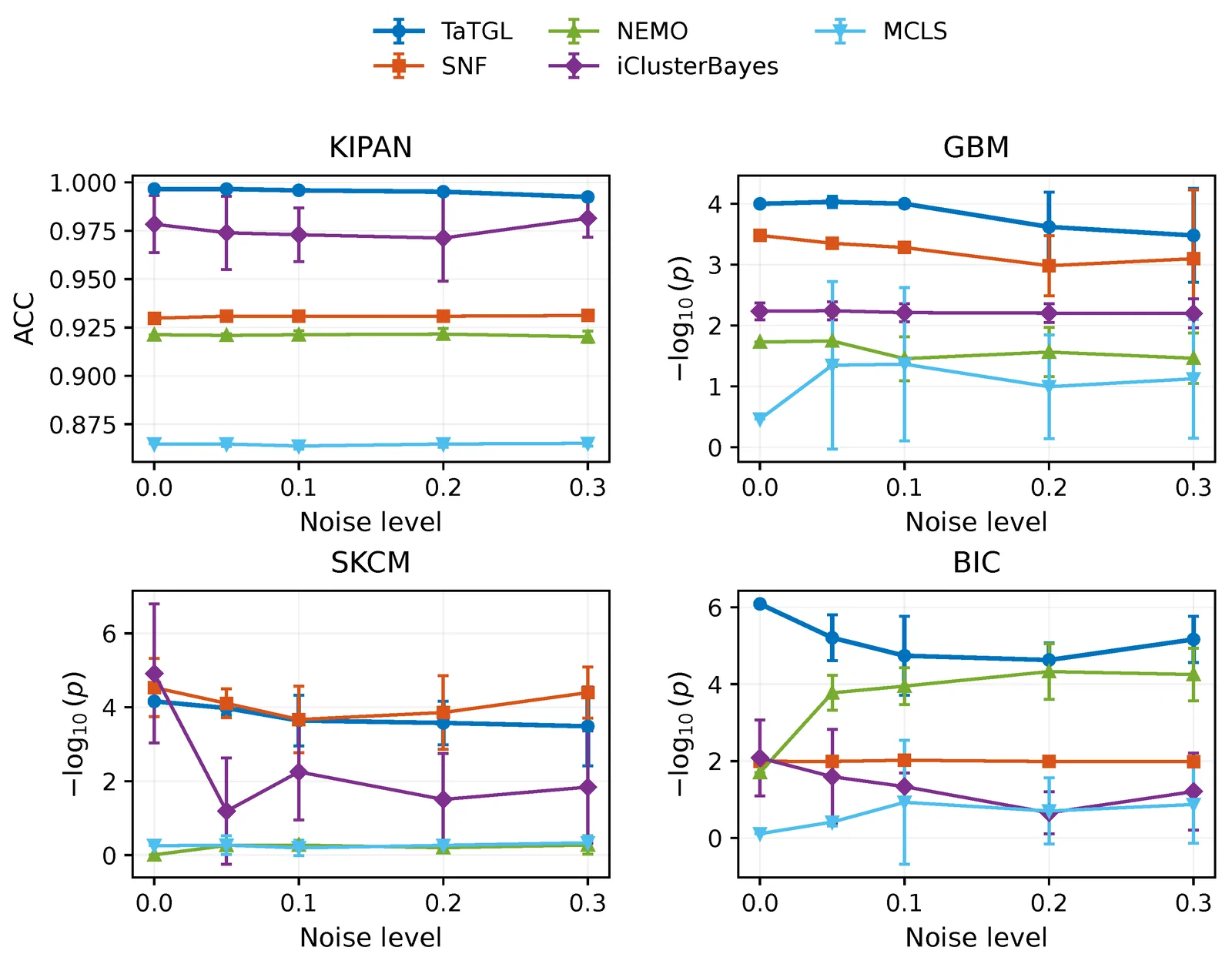
Noise robustness comparison on KIPAN, GBM, SKCM, and BIC. Zero-mean Gaussian noise is added to each omics feature with a scale proportional to the feature-wise standard deviation. Curves report the mean over five independent runs, and error bars indicate sample standard deviation.

**Figure 5:**
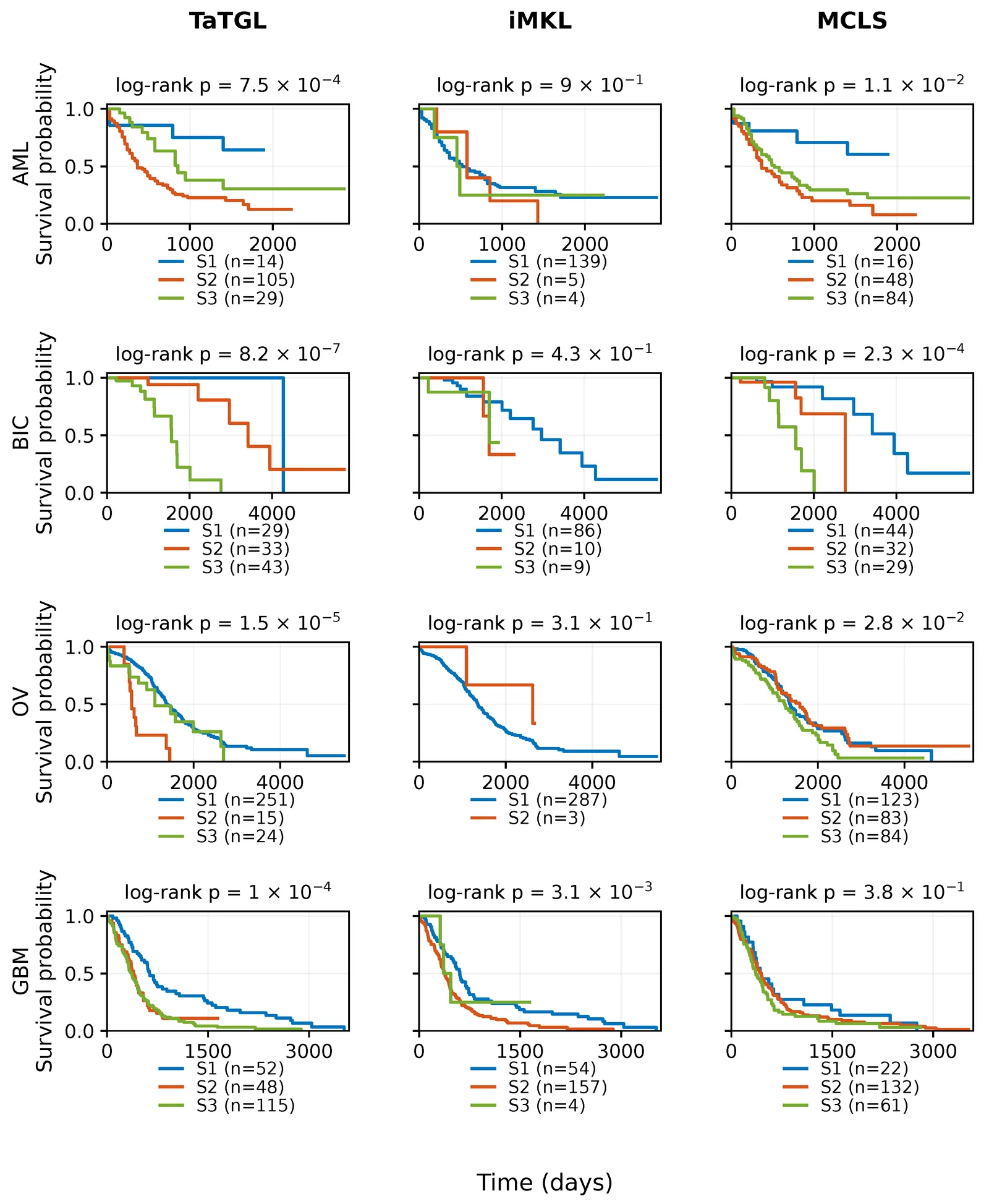
Kaplan–Meier survival analysis of the subtypes discovered by TaTGL, iMKL, and MCLS on AML, BIC, OV, and GBM datasets. Rows correspond to datasets and columns to methods. Legends indicate subtypes and sample counts; log-rank *p*-values appear above each panel.

**Figure 6:**
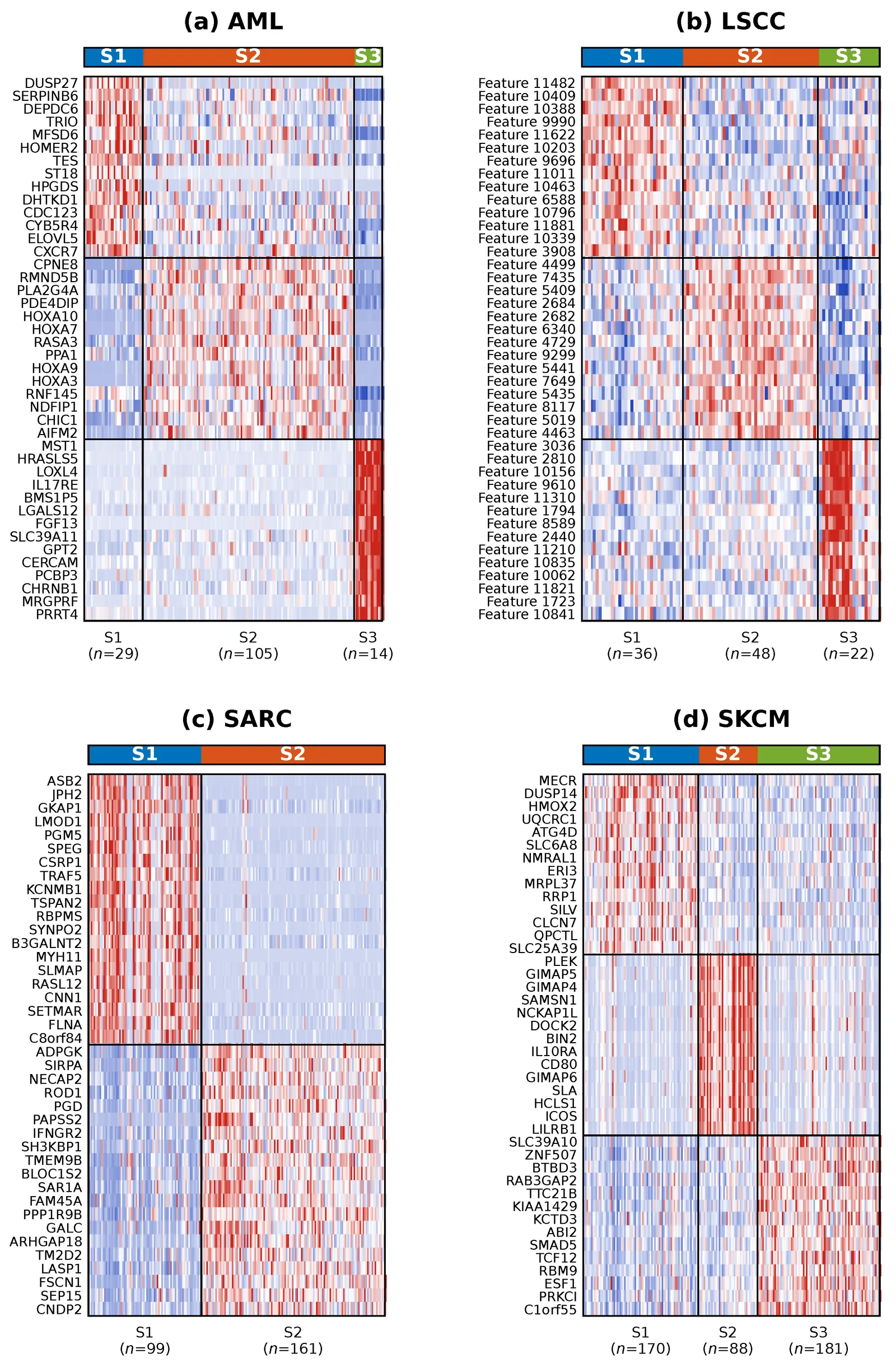
Representative subtype-associated expression heatmaps for (**a**) AML, (**b**) LSCC, (**c**) SARC, and (**d**) SKCM. Rows represent selected gene-expression features, and columns represent patients ordered by their TaTGL subtype assignments. Heatmap colors indicate row-wise z-scored expression, with red indicating higher values and blue indicating lower values. Top annotation bars identify subtypes: blue for S1, orange for S2, and green for S3, where present; *n* denotes the number of patients in each subtype. Black vertical lines separate subtypes, and black horizontal lines separate selected feature blocks. Gene symbols are shown for AML, SARC, and SKCM; LSCC labels are matrix feature indices, not quantities, because matching gene-name annotations are unavailable. These features provide descriptive characterization rather than independently validated biomarkers.

**Figure 7:**
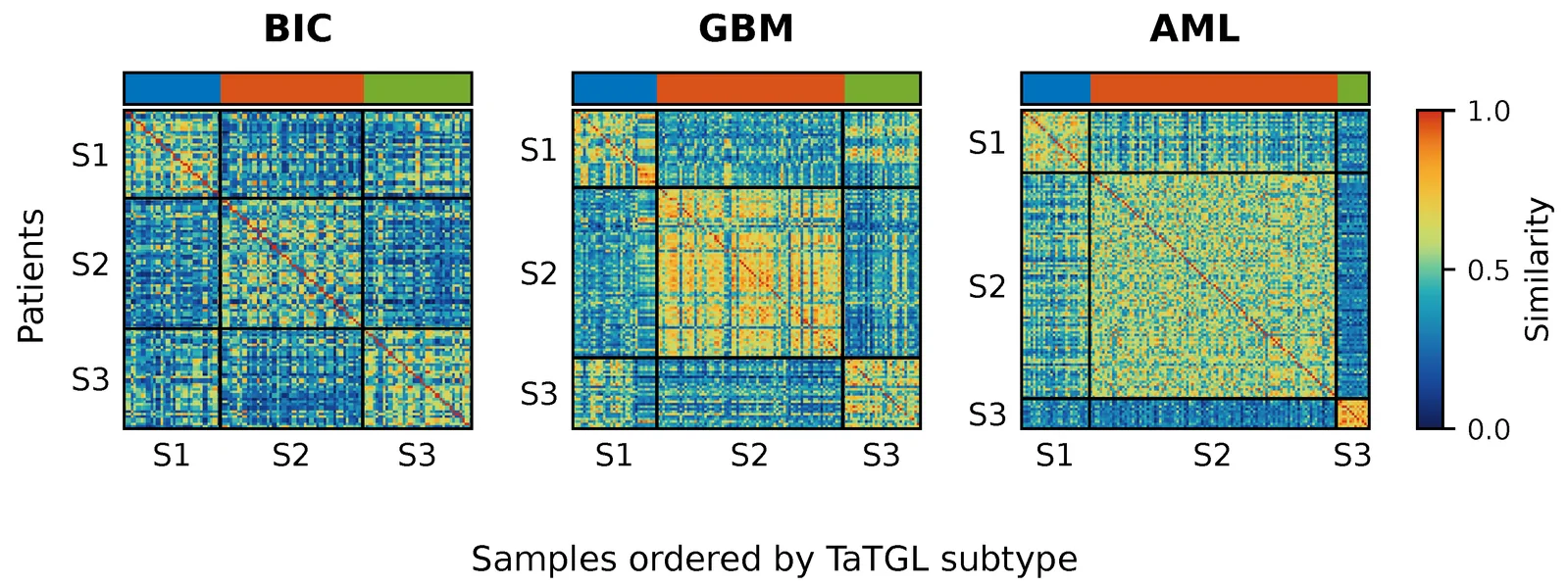
Subtype-aware patient consensus heatmaps on BIC, GBM, and AML. Samples are ordered according to the subtypes discovered by TaTGL, and the top color bars indicate subtype assignments.

**Figure 8:**
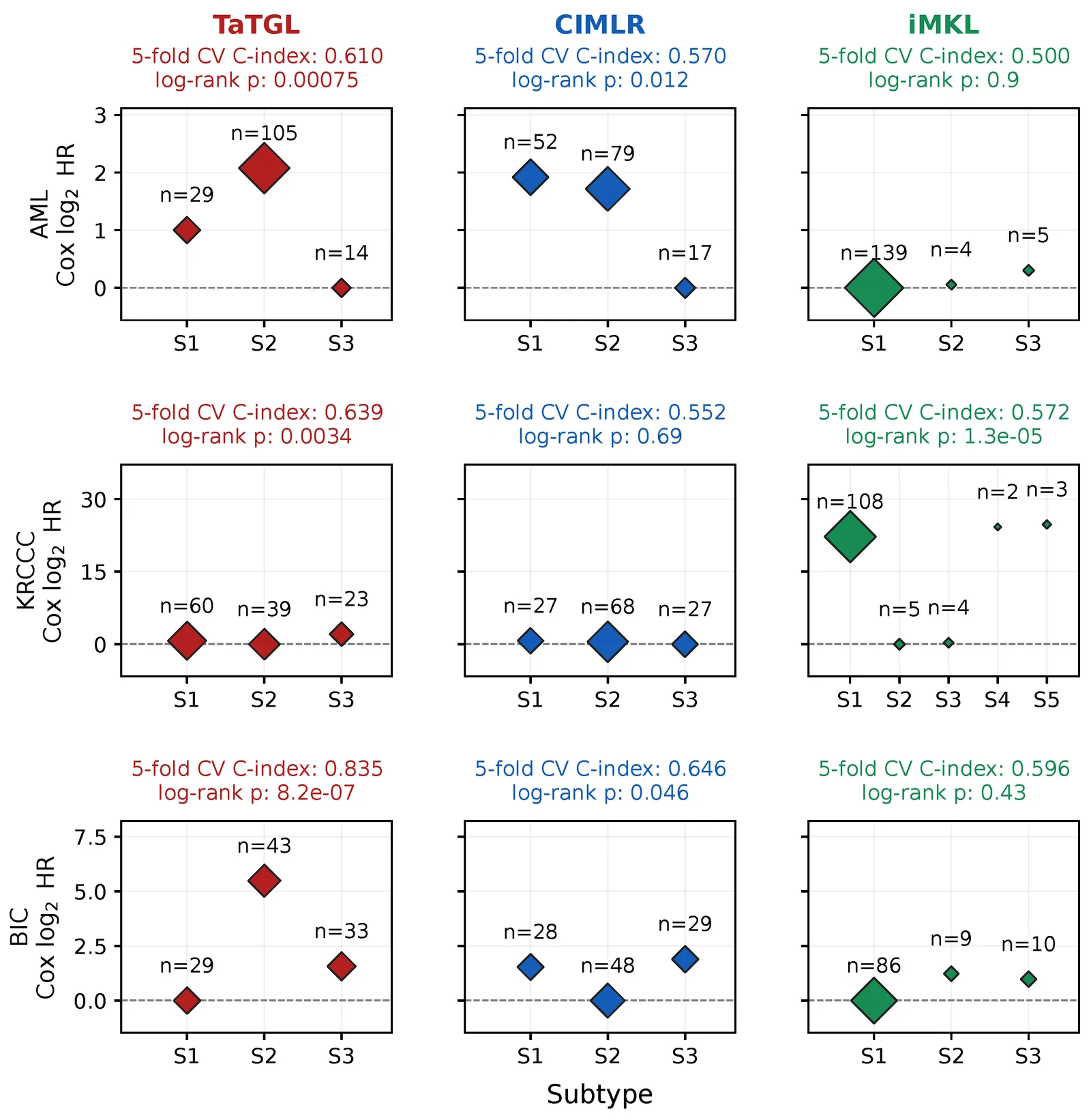
Cox subtype hazard-ratio analysis on AML, KRCCC, and BIC. Rows correspond to datasets and columns correspond to TaTGL, CIMLR, and iMKL. Each diamond denotes the subtype-level Cox $\log_{2}$ hazard ratio relative to the lowest-risk subtype within the same method, and marker size is proportional to the subtype sample count. The metric band above each panel reports the five-fold cross-validated Harrell C-index and the descriptive log-rank *p*-value.

**Table 2:** Summary of the multi-omics datasets used in the experiments.

Dataset	Setting	Samples	Reference Subtype Count	View 1	View 2	View 3
BRCA	Labeled clustering	875	5	1000	503	1000
KIPAN	Labeled clustering	584	3	1485	285	15122
AML	Survival clustering	148	No ground-truth labels	20,531	5000	705
BIC	Survival clustering	105	No ground-truth labels	17,814	23,094	354
COAD	Survival clustering	92	No ground-truth labels	17,814	23,088	312
GBM	Survival clustering	215	No ground-truth labels	12,042	1305	534
KRCCC	Survival clustering	122	No ground-truth labels	17,899	24,960	329
LIHC	Survival clustering	404	No ground-truth labels	20,531	5000	1046
LSCC	Survival clustering	106	No ground-truth labels	12,042	23,074	352
OV	Survival clustering	290	No ground-truth labels	20,531	5000	705
SARC	Survival clustering	260	No ground-truth labels	20,531	5000	1046
SKCM	Survival clustering	439	No ground-truth labels	20,531	5000	1046

Note: “No ground-truth labels” indicates that reference subtype labels and a corresponding subtype count are unavailable in the survival datasets used here. The selected cluster number supplied to each algorithm is reported separately in Table 3.

**Table 3:** Selected cluster numbers for TaTGL and the compared methods on the ten survival cohorts. Selection is based on the minimum log-rank *p*-value.

Method	AML	BIC	COAD	GBM	KRCCC	LIHC	LSCC	OV	SARC	SKCM
SNF	3	3	2	3	3	3	3	3	2	3
CIMLR	3	3	2	3	3	3	3	3	2	3
iClusterBayes	3	3	2	3	3	3	3	3	2	3
NEMO	3	3	2	3	3	3	3	3	2	3
MCLS	3	3	2	3	3	3	3	3	2	3
hMKL	3	3	2	3	5	3	3	2	2	3
iMKL	3	3	2	3	5	3	3	2	2	3
MLMF	3	3	2	3	3	3	3	3	2	3
SIMKF	3	3	2	3	5	3	3	2	2	3
TaTGL	3	3	2	3	3	3	3	3	2	3

**Table 4:** Clustering performance on labeled multi-omics cancer datasets. Results are reported as mean ± sample standard deviation. The best result on each metric is highlighted in bold, and the second-best result is underlined.

Method	BRCA				KIPAN		
	ACC	NMI	Purity		ACC	NMI	Purity
SNF	$\underline{0.6206\pm 0.0000}$	0.3390±0.0000	0.6663±0.0000		0.9298±0.0000	0.7316±0.0000	0.9298±0.0000
CIMLR	0.5177±0.0000	0.3156±0.0000	0.6491±0.0000		0.8664±0.0000	0.6319±0.0000	0.8664±0.0000
iClusterBayes	0.6053±0.0348	$\underline{0.3394\pm 0.0424}$	0.6624±0.0152		$\underline{0.9836\pm 0.0109}$	$\underline{0.9090\pm 0.0391}$	$\underline{0.9836\pm 0.0109}$
NEMO	0.5234±0.0000	0.3444±0.0000	0.6526±0.0000		0.9212±0.0000	0.7161±0.0000	0.9212±0.0000
MCLS	0.5177±0.0000	0.3146±0.0000	0.6629±0.0000		0.8630±0.0000	0.5570±0.0000	0.8630±0.0000
hMKL	0.4025±0.0280	0.2123±0.0281	0.5854±0.0190		0.5469±0.0134	0.3560±0.0261	0.7164±0.0266
iMKL	0.4937±0.0000	0.0024±0.0000	0.4983±0.0000		0.5394±0.0000	0.2749±0.0000	0.5394±0.0000
MLMF	0.4377±0.0211	0.2233±0.0248	0.6224±0.0105		0.5890±0.0640	0.1956±0.0300	0.6675±0.0375
SIMKF	0.4811±0.0000	0.0261±0.0000	0.5109±0.0000		0.4555±0.0000	0.0153±0.0000	0.4795±0.0000
TaTGL	0.6503±0.0016	0.3024±0.0035	$\underline{0.6642\pm 0.0005}$		0.9966±0.0000	0.9760±0.0000	0.9966±0.0000

**Table 5:** Survival analysis results on unlabeled multi-omics cancer datasets I. The log-rank *p*-values are reported as mean ± sample standard deviation, where smaller mean values indicate more significant survival differences among the discovered subtypes. The best result on each dataset is highlighted in bold, and the second-best result is underlined.

Method	Survival Datasets I				
	AML	BIC	COAD	GBM	KRCCC
SNF	$\left(2.66\pm 0.00\right)\times 10^{-3}$	$\left(1.01\pm 0.00\right)\times 10^{-2}$	$\left(1.38\pm 0.00\right)\times 10^{-1}$	$\left(3.31\pm 0.00\right)\times 10^{-4}$	$\left(3.69\pm 0.00\right)\times 10^{-1}$
CIMLR	$\left(1.22\pm 0.00\right)\times 10^{-2}$	$\left(4.57\pm 0.00\right)\times 10^{-2}$	$\left(9.36\pm 0.00\right)\times 10^{-3}$	$\left(4.76\pm 0.00\right)\times 10^{-3}$	$\left(6.93\pm 0.00\right)\times 10^{-1}$
iClusterBayes	$\left(1.90\pm 0.31\right)\times 10^{-2}$	$\left(2.57\pm 2.27\right)\times 10^{-1}$	$\left(1.68\pm 1.61\right)\times 10^{-1}$	$\left(6.95\pm 2.43\right)\times 10^{-3}$	$\left(2.24\pm 1.52\right)\times 10^{-1}$
NEMO	$\left(2.36\pm 0.00\right)\times 10^{-2}$	$\left(1.98\pm 0.00\right)\times 10^{-2}$	$\left(1.96\pm 0.00\right)\times 10^{-2}$	$\left(1.87\pm 0.00\right)\times 10^{-2}$	$\left(1.34\pm 0.00\right)\times 10^{-1}$
MCLS	$\left(1.11\pm 0.00\right)\times 10^{-2}$	$\left(1.70\pm 0.00\right)\times 10^{-4}$	$\left(3.74\pm 0.00\right)\times 10^{-1}$	$\left(3.73\pm 0.00\right)\times 10^{-1}$	$\left(9.51\pm 0.00\right)\times 10^{-1}$
hMKL	$\left(1.02\pm 0.45\right)\times 10^{-1}$	$\left(9.38\pm 0.01\right)\times 10^{-1}$	$\left(2.39\pm 0.28\right)\times 10^{-1}$	$\left(2.51\pm 0.08\right)\times 10^{-2}$	$\left(5.22\pm 1.91\right)\times 10^{-1}$
iMKL	$\left(8.98\pm 0.00\right)\times 10^{-1}$	$\left(4.27\pm 0.00\right)\times 10^{-1}$	$\left(5.59\pm 0.00\right)\times 10^{-1}$	$\left(3.13\pm 0.02\right)\times 10^{-3}$	$\left(2.39\pm 3.99\right)\times 10^{-1}$
MLMF	$\left(9.52\pm 0.00\right)\times 10^{-1}$	$\left(6.28\pm 0.00\right)\times 10^{-1}$	$\left(6.54\pm 0.00\right)\times 10^{-1}$	$\left(1.41\pm 0.00\right)\times 10^{-1}$	$\left(3.08\pm 0.00\right)\times 10^{-1}$
SIMKF	$\left(4.76\pm 2.84\right)\times 10^{-1}$	$\left(3.21\pm 3.15\right)\times 10^{-1}$	$\left(4.61\pm 1.80\right)\times 10^{-1}$	$\left(3.49\pm 1.64\right)\times 10^{-1}$	$\left(2.91\pm 2.95\right)\times 10^{-1}$
TaTGL	$\left(7.46\pm 0.00\right)\times 10^{-4}$	$\left(8.19\pm 0.00\right)\times 10^{-7}$	$\left(1.47\pm 0.00\right)\times 10^{-2}$	$\left(1.00\pm 0.00\right)\times 10^{-4}$	$\left(8.36\pm 10.19\right)\times 10^{-3}$

**Table 6:** Survival analysis results on unlabeled multi-omics cancer datasets II. The log-rank *p*-values are reported as mean ± sample standard deviation, where smaller mean values indicate more significant survival differences among the discovered subtypes. The best result on each dataset is highlighted in bold, and the second-best result is underlined.

Method	Survival Datasets II				
	LIHC	LSCC	OV	SARC	SKCM
SNF	$\left(2.20\pm 0.00\right)\times 10^{-1}$	$\left(2.10\pm 0.00\right)\times 10^{-3}$	$\left(2.47\pm 0.00\right)\times 10^{-1}$	$\left(1.30\pm 0.00\right)\times 10^{-1}$	$\left(7.71\pm 11.35\right)\times 10^{-5}$
CIMLR	$\left(3.23\pm 0.00\right)\times 10^{-3}$	$\left(4.77\pm 0.00\right)\times 10^{-2}$	$\left(8.50\pm 0.00\right)\times 10^{-1}$	$\left(4.75\pm 0.00\right)\times 10^{-1}$	$\left(5.08\pm 0.00\right)\times 10^{-1}$
iClusterBayes	$\left(2.00\pm 3.45\right)\times 10^{-1}$	$\left(1.40\pm 1.45\right)\times 10^{-1}$	$\left(3.08\pm 2.93\right)\times 10^{-1}$	$\left(2.91\pm 2.83\right)\times 10^{-1}$	$\left(9.19\pm 12.53\right)\times 10^{-3}$
NEMO	$\left(3.67\pm 0.00\right)\times 10^{-3}$	$\left(3.88\pm 0.00\right)\times 10^{-2}$	$\left(8.89\pm 0.07\right)\times 10^{-1}$	$\left(3.12\pm 0.00\right)\times 10^{-1}$	$\left(9.83\pm 0.00\right)\times 10^{-1}$
MCLS	$\left(2.78\pm 0.00\right)\times 10^{-2}$	$\left(1.11\pm 0.00\right)\times 10^{-3}$	$\left(2.44\pm 0.00\right)\times 10^{-2}$	$\left(4.14\pm 0.00\right)\times 10^{-3}$	$\left(3.72\pm 0.00\right)\times 10^{-1}$
hMKL	$\left(5.00\pm 0.00\right)\times 10^{-2}$	$\left(5.95\pm 0.71\right)\times 10^{-1}$	$\left(6.32\pm 0.00\right)\times 10^{-2}$	$\left(8.93\pm 0.94\right)\times 10^{-1}$	$\left(2.01\pm 0.02\right)\times 10^{-1}$
iMKL	$\left(7.30\pm 0.00\right)\times 10^{-1}$	$\left(3.39\pm 0.00\right)\times 10^{-1}$	$\left(2.94\pm 0.26\right)\times 10^{-1}$	$\left(8.20\pm 0.00\right)\times 10^{-1}$	$\left(1.47\pm 0.00\right)\times 10^{-1}$
MLMF	$\left(5.38\pm 0.00\right)\times 10^{-1}$	$\left(7.62\pm 0.00\right)\times 10^{-1}$	$\left(9.03\pm 0.00\right)\times 10^{-1}$	$\left(5.37\pm 0.00\right)\times 10^{-1}$	$\left(3.76\pm 0.00\right)\times 10^{-3}$
SIMKF	$\left(8.73\pm 2.52\right)\times 10^{-1}$	$\left(5.70\pm 3.25\right)\times 10^{-1}$	$\left(5.59\pm 3.11\right)\times 10^{-1}$	$\left(4.82\pm 3.63\right)\times 10^{-2}$	$\left(4.76\pm 2.17\right)\times 10^{-1}$
TaTGL	$\left(6.31\pm 2.10\right)\times 10^{-5}$	$\left(2.36\pm 0.00\right)\times 10^{-3}$	$\left(1.52\pm 0.00\right)\times 10^{-5}$	$\left(2.84\pm 0.00\right)\times 10^{-2}$	$\left(6.91\pm 0.00\right)\times 10^{-5}$

**Table 7:** Number of survival datasets with statistically significant subtype separation among the ten unlabeled survival cohorts. A dataset is counted as significant when its mean log-rank *p*-value over five independent runs is smaller than 0.05. The largest count is highlighted in bold.

Metric	Compared Methods									
	SNF	CIMLR	iCluster Bayes	NEMO	MCLS	hMKL	iMKL	MLMF	SIMKF	TaTGL
Count	5	6	3	6	6	1	1	1	1	**10**

**Table 8:** Statistical comparison across all reported evaluation tasks. Average ranks are computed from the mean results across six labeled clustering metrics and ten survival-clustering tasks; lower values indicate better overall performance. The Wilcoxon column reports one-sided paired signed-rank *p*-values comparing TaTGL against each method using task-wise ranks.

Method	Avg. Rank	Median Rank	Best	Second Best	*p* vs. TaTGL
TaTGL	1.63	1.00	11	3	–
SNF	3.25	3.00	1	5	0.0075
iClusterBayes	4.13	4.50	0	4	0.0021
NEMO	4.69	4.00	1	1	0.0024
MCLS	4.91	5.25	2	2	0.0022
CIMLR	5.28	5.00	1	1	0.0006
hMKL	7.44	7.50	0	0	0.0002
iMKL	7.56	8.50	0	0	0.0002
MLMF	8.06	8.00	0	0	0.0002
SIMKF	8.06	8.00	0	0	0.0002

**Table 9:** Ablation study on labeled cancer subtype datasets. Higher ACC, NMI, and Purity indicate better clustering performance. The best result on each metric is highlighted in bold.

Method	BRCA				KIPAN		
	ACC	NMI	Purity		ACC	NMI	Purity
TaTGL	**0.6503**	0.3024	**0.6642**		**0.9966**	**0.9760**	**0.9966**
TaTGL-w/o-FW-tSVD	0.6309	0.3311	0.6606		0.9914	0.9464	0.9914
TaTGL-w/o-TPGL	0.6046	**0.3727**	0.6480		0.9897	0.9369	0.9897
TaTGL-w/o-LocalManifold	0.5040	0.2928	0.6446		0.9161	0.7430	0.9161
TaTGL-w/o-Tensor	0.6217	0.3091	0.6640		0.9230	0.7138	0.9230

**Table 10:** Ablation study on unlabeled survival datasets. Lower log-rank *p*-value indicates stronger survival separation. The best result on each dataset is highlighted in bold.

Method	LIHC	BIC	SKCM
TaTGL	$6.3136\times 10^{-5}$	$8.1869\times 10^{-7}$	$6.9097\times 10^{-5}$
TaTGL-w/o-FW-tSVD	$8.9964\times 10^{-5}$	$1.7105\times 10^{-4}$	$1.7648\times 10^{-4}$
TaTGL-w/o-TPGL	$2.1313\times 10^{-4}$	$3.8800\times 10^{-4}$	$9.5904\times 10^{-5}$
TaTGL-w/o-LocalManifold	$2.7003\times 10^{-3}$	$2.7990\times 10^{-2}$	$6.9584\times 10^{-5}$
TaTGL-w/o-Tensor	$5.4347\times 10^{-1}$	$1.4551\times 10^{-2}$	$1.0991\times 10^{-2}$

## Data Availability

The datasets used in this study are publicly available from the sources cited in the manuscript. The preprocessed data for the ten survival cohorts, including gene expression, DNA methylation, miRNA expression, and patient survival data, are available from the Multi-Omic Cancer Benchmark at https://acgt.cs.tau.ac.il/multi_omic_benchmark/download.html. (accessed on 16 September 2026) The source code and processed demonstration data are available at https://github.com/hanruiTheKeter/Demo_TaTGL. (accessed on 16 September 2026)

## Abbreviations

The following abbreviations are used in this manuscript:

ADMM	Alternating direction method of multipliers
ACC	Accuracy
CNV	Copy number variation
KM	Kaplan–Meier
NMI	Normalized mutual information
SNF	Similarity network fusion
TaTGL	Topology-aware tensor graph learning
t-SVD	Tensor singular value decomposition
